# Hájek–Rényi inequality for *m*-asymptotically almost negatively associated random vectors in Hilbert space and applications

**DOI:** 10.1186/s13660-018-1671-5

**Published:** 2018-04-10

**Authors:** Mi-Hwa Ko

**Affiliations:** Division of Mathematics and Informational Statistics, Wonkwang University, Jeonbuk, Korea

**Keywords:** 60F15, Asymptotically almost negative association, Hilbert space, Hájek–Rényi inequality, Strong law of large numbers, Mixing coefficients

## Abstract

In this paper, we obtain the Hájek–Rényi inequality and, as an application, we study the strong law of large numbers for *H*-valued *m*-asymptotically almost negatively associated random vectors with mixing coefficients $\{q(n), n\geq1\}$ such that $\sum_{n=1}^{\infty}q(n)^{2}<\infty$.

## Introduction

A finite sequence $\{X_{1}, \ldots, X_{n}\}$ of $\mathbb{R}^{d}$-valued random vectors is said to be negatively associated (NA) if for any disjoint subsets $A, B \subset\{1,2,\dots, n\}$ and any real coordinatewise nondecreasing functions *f* on $\mathbb{R}^{ \vert A \vert d}$ and *g* on $\mathbb{R}^{ \vert B \vert d}$,

1.1 $$ \operatorname{Cov}\bigl(f(X_{i}, i\in A), g(X_{j}, j\in B)\bigr)\leq0, $$

whenever the covariance exists, where $\vert A \vert $ denotes the cardinality of a set *A*. An infinite sequence $\{X_{n}, n\geq1\}$ of $\mathbb {R}^{d}$-valued random vectors is NA if every finite subsequence is NA. This definition was introduced by Ko et al. [1], and in the case $d=1$ the concept of negative association was introduced by Joag-Dev and Proschan [2].

Let *H* be a real separable Hilbert space with the norm $\Vert \cdot \Vert $ generated by an inner product $\langle\cdot,\cdot\rangle$. Let $\{ e_{j}, j\geq1\}$ be an orthonormal basis in *H*. For an *H*-valued random vector *X*, we denote $X^{(j)}=\langle X, e_{j}\rangle$.

Ko et al. [1] also extended the concept of NA for $\mathbb {R}^{d}$-valued random vectors to random vectors with values in a real separable Hilbert space as follows: A sequence $\{X_{n}, n\geq1\}$ of random vectors taking values in a real separable Hilbert space $(H, \langle\cdot,\cdot\rangle)$ is called NA if for some orthonormal basis $\{e_{k}, k\geq1\}$ in *H* and for any $d\geq1$, the *d*-dimensional sequence $\{(\langle X_{n}, e_{1}\rangle, \dots, \langle X_{n}, e_{d}\rangle), n\geq1\}$ is NA.

The definitions of NA random vectors in $\mathbb{R}^{d}$ and in a Hilbert space can be applied to asymptotically almost negative association (AANA).

### Definition 1.1

A sequence $\{X_{1},\ldots,X_{n}\}$ of $\mathbb{R}^{d}$-valued random vectors is said to be AANA if there exists a nonnegative sequence $q(n)\rightarrow0$ as $n\rightarrow\infty$ such that

$$\begin{aligned} & \operatorname{Cov}\bigl(f(X_{n}),g(X_{n+1},X_{n+2}, \ldots, X_{n+k})\bigr) \\ &\quad \leq q(n) \bigl(\operatorname{Var}\bigl(f(X_{n})\bigr)\operatorname{Var}\bigl(g(X_{n+1},X_{n+2}, \ldots,X_{n+k})\bigr)\bigr)^{\frac {1}{2}} \end{aligned}$$

for all $n, k\geq1$ and all coordinatewise nondecreasing continuous functions *f* and *g* whenever the variances exist.

In the case $d=1$, the concept of AANA was introduced by Chandra and Ghosal [3, 4]. Obviously, AANA random variables contain independent random variables (with $q(n)=0$ for $n\geq1$) and NA random variables. Chandra and Ghosal [3] pointed out that NA implies AANA, but AANA does not imply NA. Because NA has been applied to the reliability theory, multivariate statistical analysis, and percolation theory, the extension of the limit properties of NA random variables to AANA random variables is of interest in theory and applications.

Since the concept of AANA was introduced, various investigations have been established by many authors. For more detail, we can refer to Chandra and Ghosal [3, 4], Ko et al. [5], Yuan and An [6, 7], Wang et al. [8], Tang [9], Shen and Wu [10], and so forth.

### Definition 1.2

A sequence $\{X_{i}, i\geq1\}$ of random vectors taking values in a real separable Hilbert space $(H, \langle\cdot,\cdot\rangle)$ is said to be AANA if for some orthonormal basis $\{e_{k}, k\geq1\}$ in *H* and for any $d\geq1$, the *d*-dimensional sequence $\{(\langle X_{i}, e_{1}\rangle,\ldots,\langle X_{i}, e_{d}\rangle), i\geq1\}$ is AANA.

We now introduce the notion of *m*-asymptotically almost negative association (*m*-AANA) in $\mathbb{R}^{d}$ and in a Hilbert space.

### Definition 1.3

Let $m\geq1$ be an integer. We say that a sequence of $\mathbb{R}^{d}$-valued random vectors $\{X_{1},\ldots ,X_{n}\}$ is *m*-asymptotically almost negatively associated (*m*-AANA) if there exists a nonnegative sequence $q(n)\rightarrow0$ as $n\rightarrow\infty$ such that

$$\begin{aligned} & \operatorname{Cov}\bigl(f(X_{n}),g(X_{n+m},\ldots, X_{n+k})\bigr) \\ &\quad \leq q(n) \bigl(\operatorname{Var}\bigl(f(X_{n})\bigr)\operatorname{Var}\bigl(g(X_{n+m}, \ldots,X_{n+k})\bigr)\bigr)^{\frac {1}{2}} \end{aligned}$$

for all $n\geq1$ and $k\geq m$ and for all coordinatewise nondecreasing continuous functions *f* and *g* whenever the variances exist.

In the case $d=1$, Nam, Hu, and Volodin [11] introduced the concept of *m*-AANA and investigated maximal inequalities and strong law of large numbers.

The family of *m*-AANA sequence contains AANA (with $m=1$), NA, and independent sequences as particular cases.

### Definition 1.4

Let $m\geq1$ be an integer. A sequence $\{X_{i}, i\geq1\}$ of random vectors taking values in a real separable Hilbert space $(H, \langle\cdot,\cdot\rangle)$ is said to be *m*-AANA if for some orthonormal basis $\{e_{k}, k\geq1\}$ in *H* and for any $d\geq1$, the *d*-dimensional sequence $\{(\langle X_{i}, e_{1}\rangle,\ldots,\langle X_{i}, e_{d}\rangle), i\geq1\}$ is *m*-AANA.

Hájek and Rényi [12] proved the following important inequality: If $\{X_{n}, n \geq1\}$ is a sequence of centered independent random variables with $EX_{n}^{2}<\infty$ and $\{b_{n} , n \geq1\}$ is a sequence of nondecreasing positive numbers, then, for any $\epsilon>0$ and positive integer $m< n$,

$$P\biggl(\max_{m\leq k \leq n}\frac{ \vert \sum_{i=1}^{k} X_{i} \vert }{b_{k}}\geq \epsilon\biggr)\leq \epsilon^{-2}\Biggl( \sum_{k=m+1}^{n} \frac{EX_{k}^{2}}{b_{k}^{2}}+\sum_{k=1}^{m} \frac{EX_{k}^{2}}{b_{m}^{2}}\Biggr). $$

Since then, this type inequality has been studied by many authors. For example, Gan [13] gave the Hájek–Rényi inequality for Banach-space-valued random variables, Liu, Gan, and Chen [14] studied the Hájek–Rényi inequality for NA random variables, Ko et al. [5] obtained the Hájek–Rényi inequality for AANA random variables, and Nam et al. [11] investigated the maximal inequalities for *m*-AANA random variables. Furthermore, Miao [15] investigated the Hájek–Rényi inequality for *H*-valued dependent random vectors with mean zero.

In this paper, we obtain the Hájek–Rényi inequality, and, as an application, we give the strong law of large numbers for *m*-AANA random vectors in a Hilbert space. In particular, we extend the results in Nam, Hu, and Volodin [11] to a Hilbert space.

## Some lemmas

We start with the property of *m*-asymptotically almost negatively associated (*m*-AANA) random variables, which can be easily obtained from the definition of *m*-AANA random variables.

### Lemma 2.1

*Let* $\{X_{n}, n\geq1\}$ *be a sequence of* *m*-*AANA random variables with mixing coefficients* $\{q(n), n\geq1\}$, *and let* $\{f_{n}, n\geq1\}$ *be a sequence of nondecreasing continuous functions*. *Then* $\{f_{n}(X_{n}), n\geq1\}$ *is still a sequence of* *m*-*AANA random variables with mixing coefficients* $\{q(n), n\geq1\}$.

### Lemma 2.2

(Yuan and An [6])

*Let* $1< p\leq2$, *and let* $\{X_{n}, n\geq1\}$ *be a sequence of AANA random variables with mixing coefficients* $\{q(n), n\geq1\}$ *and* $EX_{n}=0$ *for all* $n\geq1$. *If* $\sum_{n=1}^{\infty}q^{2}(n)<\infty$, *then there exists a positive constant* $C=C(p)$ *depending only on* *p* *such that*, *for all* $n\geq1$,

2.1 $$ E\Biggl(\max_{1\leq k\leq n} \Biggl\vert \sum _{i=1}^{k} X_{i} \Biggr\vert ^{p}\Biggr) \leq C\Biggl(\sum_{i=1}^{n} E \vert X_{i} \vert ^{p}\Biggr). $$

Nam et al. [11] extended Lemma 2.2 to the case of *m*-AANA sequence.

### Lemma 2.3

(Nam et al. [11])

*Let* $1< p\leq2$, *and let* $m\geq1$ *be an integer*. *Let* $\{X_{n}, n\geq1\}$ *be a sequence of mean*-*zero* *m*-*AANA random variables with mixing coefficients* $\{ q(n), n\geq1\}$. *If* $\sum_{n=1}^{\infty}q^{2}(n)<\infty$, *then there exists a positive constant* $C=C(p)$ *depending only on* *p* *such that*

2.2 $$ E\Biggl(\max_{1\leq k\leq n} \Biggl\vert \sum _{i=1}^{k} X_{i} \Biggr\vert ^{p}\Biggr) \leq C m^{p-1}\Biggl(\sum _{i=1}^{n} E \vert X_{i} \vert ^{p}\Biggr). $$

From Lemma 2.3 we obtain the Rosenthal-type inequality for *m*-AANA random vectors with coefficients $\{q(n), n\geq1\}$ such that $\sum_{n=1}^{\infty}q^{2}(n)<\infty$ in a Hilbert space.

### Lemma 2.4

*Let* $m\geq1$ *be an integer*, *and let* $\{ X_{n}, n\geq1\}$ *be a sequence of* *H*-*valued* *m*-*AANA random vectors with mixing coefficients* $\{q(n), n\geq1\}$ *and* $EX_{n}=0$ *for all* $n\geq1$. *If* $\sum_{n=1}^{\infty}q^{2}(n)<\infty$, *then there exists a positive constant* *C* *such that*, *for all* $n\geq1$,

2.3 $$ E\Biggl(\max_{1\leq k\leq n} \Biggl\Vert \sum _{i=1}^{k} X_{i} \Biggr\Vert ^{2}\Biggr) \leq Cm\Biggl(\sum_{i=1}^{n} E \Vert X_{i} \Vert ^{2}\Biggr). $$

### Proof

From Lemma 2.3 we obtain

$$\begin{aligned} E\max_{1\leq k\leq n} \Biggl\Vert \sum_{i=1}^{k} X_{i} \Biggr\Vert ^{2} =& E\max_{1\leq k\leq n} \sum_{j=1}^{\infty}\Biggl\vert \Biggl\langle \sum_{i=1}^{k} X_{i}, e_{j}\Biggr\rangle \Biggr\vert ^{2} \\ \leq& \sum_{j=1}^{\infty}E\max _{1\leq k\leq n} \Biggl\vert \Biggl\langle \sum _{i=1}^{k} X_{i}, e_{j}\Biggr\rangle \Biggr\vert ^{2} \\ =& \sum_{j=1}^{\infty}E\max _{1\leq k\leq n} \Biggl\vert \sum_{i=1}^{k} \langle X_{i}, e_{j}\rangle \Biggr\vert ^{2} \\ \leq& Cm\sum_{j=1}^{\infty}\sum _{i=1}^{n} E \bigl\vert \langle X_{i}, e_{j}\rangle \bigr\vert ^{2}\quad \bigl(\mbox{by (2.2)}\bigr) \\ =& Cm\Biggl(\sum_{i=1}^{n} E \Vert X_{i} \Vert ^{2}\Biggr). \end{aligned}$$

 □

### Remark

From Lemma 2.4 we obtain the following Kolmogorov-type inequality for a sequence $\{X_{n}, n\geq1\}$ of mean-zero *H*-valued *m*-AANA random vectors with mixing coefficients $\{q(n), n\geq1\}$ such that $\sum_{n=1}^{\infty}q^{2}(n)<\infty$:

2.4 $$ P\Biggl(\max_{1\leq k\leq n} \Biggl\Vert \sum _{i=1}^{k} X_{i} \Biggr\Vert >\epsilon \Biggr) \leq C m\epsilon ^{-2}\sum_{i=1}^{n} E \Vert X_{i} \Vert ^{2}. $$

## Main results

### Theorem 3.1

*Let* $\{X_{n}, n\geq1\}$ *be a sequence of* *H*-*valued* *m*-*AANA random vectors with mixing coefficients* $\{q(n), n\geq1\}$ *such that* $\sum_{n=1}^{\infty}q^{2}(n)<\infty$, $EX_{n}=0$, *and* $E \Vert X_{n} \Vert ^{2}<\infty$ *for* $n\geq1$. *If* $\{b_{n}, n\geq1\}$ *is a nondecreasing sequence of positive real numbers*, *then for any* $\epsilon >0$, *we have*

3.1 $$ P\Biggl(\max_{1\leq k\leq n} \Biggl\Vert \frac{1}{b_{k}}\sum_{i=1}^{k} X_{i} \Biggr\Vert >\epsilon\Biggr) \leq 4mC\sum _{i=1}^{n} \frac{E \Vert X_{i} \Vert ^{2}}{\epsilon^{2} b_{i}^{2}}. $$

### Proof

Let $S_{n}=\sum_{i=1}^{n} X_{i}$. Without loss of generality, setting $b_{0}=0$, we have

$$\begin{aligned} S_{k} =&\sum_{j=1}^{k} b_{j}\cdot\frac{X_{j}}{b_{j}}=\sum_{j=1}^{k} \Biggl(\sum_{i=1}^{j}(b_{i}-b_{i-1}) \frac{X_{j}}{b_{j}}\Biggr) \\ =&\sum_{i=1}^{k}(b_{i}-b_{i-1}) \sum_{i\leq j\leq k}\frac{X_{j}}{b_{j}}. \end{aligned}$$

Since $(\frac{1}{b_{k}})\sum_{j=1}^{k}(b_{j}-b_{j-1})=1$, we have

3.2 $$\begin{aligned} \max_{1\leq k\leq n}\frac{1}{b_{k}} \Vert S_{k} \Vert \leq& \max_{1\leq k\leq n}\max _{1\leq i\leq k} \biggl\Vert \sum_{i\leq j\leq k} \frac{X_{j}}{b_{j}} \biggr\Vert \\ \leq& \max_{1\leq i\leq k\leq n} \biggl\Vert \sum _{j\leq k}\frac{X_{j}}{b_{j}}-\sum_{j< i} \frac{X_{j}}{b_{j}} \biggr\Vert \\ \leq& 2\max_{1\leq i\leq n} \Biggl\Vert \sum _{j=1}^{i}\frac{X_{j}}{b_{j}} \Biggr\Vert . \end{aligned}$$

Since $\{\frac{X_{j}}{b_{j}}, j\geq1\}$ is still a sequence of *H*-valued *m*-AANA random vectors with mixing coefficients $\{q(n), n\geq1\}$ such that $\sum_{n=1}^{\infty}q^{2}(n)<\infty$, by Lemma 2.1 it follows from (3.2), Markov inequality, and Lemma 2.4 that

3.3 $$ P\biggl(\max_{1\leq k\leq n}\frac{1}{b_{k}} \Vert S_{k} \Vert \geq\epsilon\biggr) \leq P\Biggl(2\max _{1\leq i\leq n} \Biggl\Vert \sum_{j=1}^{i} \frac{X_{j}}{b_{j}} \Biggr\Vert \geq\epsilon\Biggr)\leq 4mC\sum _{i=1}^{n} \frac{E \Vert X_{i} \Vert ^{2}}{\epsilon^{2} b_{i}^{2}}. $$

 □

### Remark

Under the conditions of Theorem 3.1, we obtain

3.4 $$ E\biggl(\max_{1\leq k\leq n} \biggl\Vert \frac{S_{k}}{b_{k}} \biggr\Vert \biggr)^{2} \leq4mC\sum _{i=1}^{n} \frac {E \Vert X_{i} \Vert ^{2}}{b_{i}^{2}}, $$

where $S_{k}=X_{1}+\cdots+X_{k}$.

### Proof

See the proof of Theorem 1 in Fazekas [16]. □

Note that by using (3.4) we can also prove Theorem 3.1.

From Theorem 3.1 we can get the following more generalized Hájek–Rényi inequality.

### Theorem 3.2

*Let* $m\geq1$ *be an integer*. *Let* $\{X_{n}, n\geq1\}$ *be a sequence of* *H*-*valued* *m*-*AANA random vectors with mixing coefficients* $\{q(n), n\geq1\}$ *such that* $\sum_{n=1}^{\infty}q^{2}(n)<\infty$, $EX_{n}=0$, *and* $E \Vert X_{n} \Vert ^{2}<\infty$ *for* $n\geq1$. *If* $\{ b_{n}, n\geq1\}$ *is a nondecreasing sequence of positive real numbers*, *then for any* $\epsilon>0$ *and positive integers* $l< n$,

3.5 $$ P\Biggl(\max_{l\leq k\leq n} \Biggl\Vert \frac{1}{b_{k}}\sum_{i=1}^{k} X_{i} \Biggr\Vert >\epsilon\Biggr) \leq \frac{16mC}{\epsilon^{2}}\Biggl( \frac{1}{b_{l}^{2}}\sum_{i=1}^{l} E \Vert X_{i} \Vert ^{2}+\sum_{i=l+1}^{n} \frac{E \Vert X_{i} \Vert ^{2}}{b_{i}^{2}}\Biggr). $$

### Proof

Observe that

$$\begin{aligned} P\Biggl(\max_{l\leq k\leq n} \Biggl\Vert \frac{1}{b_{k}}\sum _{i=1}^{k} X_{i} \Biggr\Vert > \epsilon\Biggr) \leq &P\biggl(\max_{l\leq k\leq n}\frac{ \Vert S_{l} \Vert }{b_{k}}> \frac{\epsilon}{2}\biggr) \\ &{}+P\biggl(\max_{l\leq k\leq n}\frac{ \Vert S_{k}-S_{l} \Vert }{b_{k}}>\frac{\epsilon}{2} \biggr) \\ =:&I_{1}+I_{2}. \end{aligned}$$

For $I_{1}$, by (3.1) of Theorem 3.1 we obtain

3.6 $$ I_{1}\leq\frac{16m C}{\epsilon^{2} b_{l}^{2}}\sum _{i=1}^{l} E \Vert X_{i} \Vert ^{2}. $$

It is clear that

3.7 $$ \max_{l\leq k\leq n}\frac{ \Vert S_{k}-S_{l} \Vert }{b_{k}}=\max _{1\leq k\leq n-l}\frac { \Vert \sum_{j=1}^{k} X_{l+j} \Vert }{b_{l+k}}. $$

For $I_{2}$, by the proof of Theorem 3.1 it follows from (3.7) that

3.8 $$ I_{2}\leq\frac{16m C}{\epsilon^{2}}\sum _{i=1}^{n-l}\frac{E \Vert X_{l+i} \Vert ^{2}}{b_{l+i}^{2}}= \frac{16mC}{\epsilon^{2}}\sum _{i=l+1}^{n} \frac{E \Vert X_{i} \Vert ^{2}}{b_{i}^{2}}. $$

Hence, by (3.6) and (3.8) we obtain (3.5). □

### Remark

Under the conditions of Theorem 3.2, we obtain

3.9 $$ E\biggl(\max_{l\leq k\leq n} \biggl\Vert \frac{S_{k}}{b_{k}} \biggr\Vert \biggr)^{2} \leq16mC\Biggl( \frac {1}{b_{l}^{2}}\sum_{i=1}^{l} E \Vert X_{i} \Vert ^{2}+\sum_{i=l+1}^{n} \frac{E \Vert X_{i} \Vert ^{2}}{b_{i}^{2}}\Biggr), $$

where $S_{k}=X_{1}+\cdots+X_{k}$.

### Proof

See the proof of Theorem 3 in Fazekas [16]. □

Note that by using (3.9) we can also prove Theorem 3.2.

## Applications

### Theorem 4.1

*Let* $\{b_{n}, n\geq1\}$ *be a nondecreasing sequence of positive numbers*. *Let* $\{X_{n}, n\geq1\}$ *be a sequence of mean*-*zero* *H*-*valued* *m*-*AANA random vectors with mixing coefficients* $\{q(n), n\geq1\}$ *such that* $\sum_{n=1}^{\infty}q^{2}(n)<\infty$. *If*

4.1 $$ \sum_{i=1}^{\infty}\frac{E \Vert X_{i} \Vert ^{2}}{b_{i}^{2}}< \infty, $$

*then*, *for* $0< r<2$,

4.2 $$ E\sup_{n\geq1}\biggl(\frac{ \Vert S_{n} \Vert }{b_{n}} \biggr)^{r}< \infty. $$

### Proof

Note that, for $0< r<2$,

$$E\sup_{n\geq1}\biggl(\frac{ \Vert S_{n} \Vert }{b_{n}}\biggr)^{r}< \infty\quad \Leftrightarrow\quad \int _{1}^{\infty}P\biggl(\sup_{n\geq1} \frac{ \Vert S_{n} \Vert }{b_{n}}>t^{\frac{1}{r}}\biggr)\,dt< \infty. $$

By (3.1) and (4.1) we get

$$\begin{aligned} \int_{1}^{\infty}P\biggl(\sup_{n\geq1} \frac{ \Vert S_{n} \Vert }{b_{n}}>t^{\frac {1}{r}}\biggr)\,dt \leq& \frac{4m C}{\epsilon^{2}} \int_{1}^{\infty}t^{-\frac {2}{r}}\sum _{n=1}^{\infty}\frac{E \Vert X_{n} \Vert ^{2}}{b_{n}^{2}} \,dt \\ =& \frac{4m C}{\epsilon^{2}} \sum_{n=1}^{\infty}\frac{E \Vert X_{n} \Vert ^{2}}{b_{n}^{2}} \int_{1}^{\infty}t^{-\frac{2}{r}} \,dt \\ < &\infty. \end{aligned}$$

 □

### Theorem 4.2

*Let* $\{b_{n}, n\geq1\}$ *be a nondecreasing unbounded sequence of positive real numbers*. *If* $\{X_{n}, n\geq1\}$ *is a sequence of* *H*-*valued* *m*-*AANA random vectors with mixing coefficients* $\{q(n), n\geq1\}$ *such that* $\sum_{n=1}^{\infty}$ $q^{2}(n)<\infty$ *and* $EX_{n}=0$. *If* (4.1) *is satisfied*, *then*

4.3 $$ \frac{S_{n}}{b_{n}}\rightarrow0\quad \textit{a.s.} $$

### Proof

By Theorem 3.2 we have

$$P\Biggl(\max_{l\leq k\leq n} \Biggl\Vert \frac{1}{b_{k}}\sum _{i=1}^{k} X_{i} \Biggr\Vert > \epsilon\Biggr)\leq \frac{16m C}{\epsilon^{2}} \Biggl(\sum_{i=l+1}^{n} \frac{E \Vert X_{i} \Vert ^{2}}{b_{i}^{2}}+\frac {1}{b_{l}^{2}}\sum_{i=1}^{l} E \Vert X_{i} \Vert ^{2}\Biggr). $$

However,

$$\begin{aligned} P\Biggl(\sup_{n\geq1} \Biggl\Vert \frac{1}{b_{n}}\sum _{i=1}^{n} X_{i} \Biggr\Vert > \epsilon\Biggr) =&\lim_{n\rightarrow\infty}P\Biggl(\max_{l\leq k\leq n} \Biggl\Vert \frac{1}{b_{k}}\sum_{i=1}^{k} X_{i} \Biggr\Vert >\epsilon\Biggr) \\ \leq& \frac{16m C}{\epsilon^{2}} \Biggl(\sum_{j=l+1}^{\infty}\frac{E \Vert X_{j} \Vert ^{2}}{b_{j}^{2}}+\sum_{j=1}^{l} \frac{E \Vert X_{j} \Vert ^{2}}{b_{l}^{2}}\Biggr). \end{aligned}$$

By the Kronecker lemma we get

$$\lim_{n\rightarrow\infty}P\Biggl(\sup_{n\geq1} \Biggl\Vert \frac{1}{b_{n}}\sum_{i=1}^{n} X_{i} \Biggr\Vert >\epsilon\Biggr)=0. $$

Hence, the proof is complete. □

### Remark

Note that Theorem 4.2 can be also proved by the Hájek–Rényi-type maximal inequality for moments (3.9) (see Theorem 2 of Fazekas [16]).

From Theorem 3.2 we have the following:

### Corollary 4.3

*Let* $m\geq1$ *be an integer*. *If* $\{ X_{n}, n\geq1\}$ *is a sequence of mean*-*zero* *H*-*valued* *m*-*AANA random vectors with mixing coefficients* $\{q(n), n\geq1\}$ *such that* $\sum_{n=1}^{\infty}q^{2}(n)<\infty$, *then*, *for any* $0< t<2$ *and* $\epsilon>0$, *we have*

4.4 $$ P\biggl(\sup_{j\geq l}\frac{ \Vert S_{j} \Vert }{j^{\frac{1}{t}}}\geq\epsilon \biggr)\leq16mC \epsilon^{-2} \frac{2}{2-t}l^{(t-2)/t}\sup _{n} E \Vert X_{n} \Vert ^{2}, $$

*where* *l* *is an arbitrary positive number*.

From Theorems 4.1 and 4.2 we have the following:

### Corollary 4.4

*Let* $m\geq1$ *be an integer*. *If* $\{ X_{n}, n\geq1\}$ *is a sequence of mean*-*zero* *H*-*valued* *m*-*AANA random vectors with mixing coefficients* $\{q(n), n\geq1\}$ *such that* $\sum_{n=1}^{\infty}q^{2}(n)<\infty$ *and* $\sup_{n}E \Vert X_{n} \Vert ^{2}<\infty$, *then*, *for any* $0< t<2$,

4.5 $$ E\sup_{n}\biggl(\frac{ \Vert S_{n} \Vert }{n^{\frac{1}{t}}} \biggr)^{r}< \infty \quad \textit{for }0< r< 2, $$

*and*

4.6 $$ \frac{S_{n}}{n^{\frac{1}{t}}}\rightarrow0\quad \textit{a.s. as }n \rightarrow \infty. $$

### Remark

If $\{X_{n}, n\geq1\}$ is a sequence of *H*-valued *m*-NA random vectors with $EX_{n}=0$ and $E \Vert X_{n} \Vert ^{2}<\infty$, then all results in Sect. 3 still hold (see Miao [15]).

## Conclusions

1. Note that 1-AANA is AANA. Hence, when $m=1$, some lemmas in Sect. 2 and the main results in Sect. 3 still hold for AANA random vectors in a Hilbert space.
2. The results in Nam, Hu, and Volodin [11] can be extended to a Hilbert space only when $p=2$.
